# Oral Microflora in the Background of Oral Cancer: A Review

**DOI:** 10.7759/cureus.33129

**Published:** 2022-12-30

**Authors:** Srajan S Hora, Swati K Patil

**Affiliations:** 1 Department of Oral Pathology and Microbiology, Sharad Pawar Dental College, Datta Meghe Institute of Higher Education and Research, Wardha, IND

**Keywords:** oral hygiene, smoking, alcohol, oral squamous cell carcinoma, microflora

## Abstract

Oral cancer exhibits a multifactorial etiology. Microorganisms residing within the oral cavity as normal commensals have long been studied in terms of their role in the process of carcinogenesis. Other factors such as tobacco and alcohol consumption have also been implicated in carcinogenesis as the primary risk factors. Poor oral hygiene, dietary abnormalities, and betel nut chewing can also act as contributory factors in the process of carcinogenesis. Multiple research works have been carried out in the past to shed some light on the role of exogenous bacterial species in the development of cancers. Studies conducted were to assess changes in the oral microflora in patients suffering from oral carcinoma and to evaluate and compare pre-operative and post-operative changes in oral microbiota. For this review, multiple articles were studied and evaluated. Appropriate conclusions were drawn and are presented in the review. A definitive link between cancer and microflora is yet to be established. In the present article, a review of the studies done on the contribution of microbial flora present within the oral cavity and their role in oral cancer is done and its nature and extent are evaluated. A variety of microbiological agents can contribute to the progression of carcinogenesis in the presence of definitive risk factors such as alcoholism and smoking.

## Introduction and background

The oral tissues and structures have a heterogenous nature, providing a complex and diverse ecological habitat to all the microorganisms that reside in various niches within the oral cavity [1]. The complexity of the oral microbiota can be attributed to the fact that the oral cavity is the only structure of the body wherein natural exposure of the mineralized tissues, i.e, the teeth to the external environment, takes place. The bacterial flora residing within the mouth is actively involved in the maintenance of oral health. Such bacterial species live in harmony with the host and are considered commensal microorganisms. A disturbance of this equilibrium and changes in the composition of microflora is known to be responsible for the initiation of various diseases that can have both systemic and oral implications. Numerous studies have been conducted both in the past and the present to shed some light on the role of external etiological factors in the process of carcinogenesis, the most controversial among them being the role of microbiota, and deleterious habits such as smoking and alcoholism [1]. Diet, nutrition status, and overall immunological status of an individual are also believed to be of great significance when it comes to the body's ability to ward off cancer-causing changes.

Oral cancer, namely oral squamous cell carcinoma (OSCC), which is among the most prevalent cancers worldwide, is one condition whose occurrence can have an association with changes in the oral microbiota. The nature and extent of this association is an area of ambiguity in terms of how and to what extent the microbiota influences the oral environment and the changes that occur leading to the development of carcinoma. In recent times, the human oral microbiome has been considered a new potential biomarker reservoir for carcinomas of the oral cavity. Many reports show a few significant species in altered tissues, such as *Pseudomonas aeruginosa* and *Fusobacterium nucleatum*, and these species are found to be linked with OSCC. Other reports show that the following five genera - *Bacillus*, *Parvimonas*, *Peptostreptococcus*, *Enterococcus,* and *Slackia -* showed differences among patients with precursor lesions of epithelial origin and OSCC [1]. The characterization of changes in oral microbiota could also help to shed some light on assessing the prophylactic drug therapy in such patients, with better accuracy. Meticulous assessment of the role of these factors in the process of carcinogenesis can help in the development of a thorough treatment plan for carcinoma patients, that will enable them to live a fulfilling life with minimum complications both during and after treatment. Therapies aimed at targeting the causative factors at their stem will enable clinicians to better understand the disease as well as its management.

## Review

Hypothesis

According to reports, persistent inflammation encourages the growth of a variety of cancers, including OSCC [1]. Gaonkar et al. studied human oral microbiota and its association with the process of carcinogenesis [1]. Their study was unable to establish any direct causative role; however, they suggested that the oral microflora may be able to act as a synergist along with the known significant risk factors, like alcoholism and smoking. Periodontitis and oral cancer are linked to some pathogenic bacteria. However, the full oral microbiome profile throughout the development of cancer from the early stage to the late stage is still a mystery [2]. In the last few decades, a lot of theories claiming a connection between cancer and bacteria, fungus, and viruses have been proposed. Dysbiosis is the term for an altered microbiome that has been linked to a disease, and it may have its roots in an "alpha bug" that takes advantage of an ecological niche (like *Porphyromonas gingivalis* and periodontal disease) and causes both inflammation and compensatory responses in the commensal microbial community. The production of chemical carcinogens like acetaldehyde (ACH) and N-nitroso compounds as well as inflammation caused by dysbiosis are just a few of the pathways that have been suggested as ways in which the microbiota may contribute to the development of cancer [3].

Given the abundance and diversity of the oral bacterial flora, thorough research would be quite difficult. There are between 300 and 400 species that can be detected, and there are undoubtedly still many more to be found [4]. Before delving into the apparent function of bacteria in carcinogenesis, it is crucial to describe the normal oral microbial flora in its entirety because there is a distinct, healthy, site-specific, and highly diversified bacterial flora of the mouth cavity. It is crucial to remember that numerous oral disorders are caused by microorganisms in the oral cavity, and there may be a connection between the two [5]. Within the body, alcohol gets converted into its first metabolic product, ACH, a definite carcinogen, via the alcohol dehydrogenase effect of some specific bacterial species, like streptococci. Some other Gram-positive and Gram-negative aerobic bacteria like *Neisseria* can also be involved in this process. Although there is no specific evidence backing the role of the bacterial population of the oral cavity in the process of carcinogenesis, there is however a possibility that these microbes play a role in the progression and advancement of oral carcinogenesis. Several different bacteria have been suggested to cause carcinogenesis, either by triggering chronic inflammation, by interfering with signaling pathways and cell cycles, or through mutagenic ACH metabolism [6]. There is not much research on the ACH generation of oral microorganisms on normal, chronically inflamed, or cancerous oral mucosal surfaces. Saliva samples or mouthwashes were used in every prior study on ACH synthesis by bacteria from the oral cavity [7]. In a study conducted by Hooper et al. (2007), it was mentioned that specific oral microbiota members were more prevalent on the surface of tumors in their research of intraoral carcinomas compared to control sites {8}. Recent reports suggest that people with OSCC typically have much higher quantities of specific microorganisms in their saliva. Due to its potential utility as a diagnostic tool, this apparent modification of the oral microbiota in instances of OSCC is of considerable interest [8].

The 20-year revolution in cancer treatment has been largely facilitated by viruses. It is difficult to imagine that the molecular foundation of cancer would stand disclosed so clearly today without the contributions of viral carcinogenesis. Despite being initially dismissed as strange agents that only caused cancer in animals and had no application to people, viruses have proven to be the key to understanding how to govern cell growth [9]. Pathogenic bacteria present in the oral cavity can act as periodontal pathogenic organisms and may further aggravate the overall disease prognosis. This indicates that there is a need for effective and optimum oral hygiene practices in such patients. The resident oral microbiota forms a commensal niche, that is non-pathogenic and contributes to the overall host immunity. But, due to changes in the ecology of the site involved during the development of cancer, a gradual change in the equilibrium of the resident microflora leads to the progression of carcinogenesis. Based on the fact that some species of Candida have higher nitrosation potential, consequently producing nitrosamine chemicals that have a role in the onset of carcinogenesis, evidence points to greater involvement for the *Candida* species in oral carcinogenesis. Literature also suggests that *Candida* may aid in the development of cancer [10]. Conversely, as a result of carcinoma, there can be an alteration in the population and behavior of the oral microbiota due to disrupted routine oral hygiene practices.

Potentially oncogenic oral bacteria

A variety of bacterial species can be isolated from the oral cavity in healthy individuals and from patients suffering from oral squamous cell carcinoma. Analysis and comparison of the species between the two groups can help gather some knowledge about bacterial species that are potentially oncogenic. Head and neck carcinomas were field-carcinogenized as a result of *Streptococcus anginosus* infection. Perhaps the normal mucosal barrier is destroyed by extrinsic factors like alcohol and tobacco, which leads to chronic inflammation and the development of *S. anginosus* infection, which aids in the development of head and neck carcinomas [11].

Mager et al. in their study on oral microflora tested 40 different species of bacteria residing within the oral cavity, found after comparing three bacterial species, namely, *Capnocytophaga gingivalis*, *Prevotella melaninogenica*, and *Streptococcus mitis*. These species were found to be elevated in salivary samples taken from patients suffering from OSCC. The study suggested that these three species can be considered diagnostic biomarkers and can be helpful in predicting carcinoma cases [12]. In studies conducted by Sakamoto et al., oral streptococcal species - *Streptococcus constellatus*, *Streptococcus oralis, S. mitis, Streptococcus sanguis, and Streptococcus salivarius -* were among the most frequent isolates from the cervical group of lymph nodes in oral cancer patients [13]. Numerous harmful bacteria alter host cell signaling pathways and thereby regulate cell behavior to improve the pathogen's chances of survival. The development or suppression of tumor formation depends heavily on the modulation of these signaling factors. Due to the requirement that infected cells grow and their resistance to the natural control systems, chronic bacterial infection with intracellular bacteria appears to be characterized by the perturbation of host signaling. Such infections have the potential to imitate some of the obtrusive features of tumorigenesis, and in fact, the pseudocancerous lesions that develop in such infections may recover following antibiotic therapy and bacterial clearance [14]. Studies on the fungi linked to oral infections after radiation have revealed that while Aspergillus spp. and *Torulopsis glabrata* are occasionally isolated, Candida spp. are typically prevalent. Antifungal drug sensitivity of such yeasts is frequently unknown, necessitating empirical therapy. Furthermore, it is unknown if radiation therapy affects how sensitive yeasts are. For the treatment of oral and other diseases, patients who get external radiotherapy frequently have side effects at the irradiation site on their skin. These typically appear as severe erythematous lesions that are dry or moist desquamative lesions. The presence of fungus has only rarely been documented in these illnesses, which are assumed to be predominately bacterial [15].

All over the world, oral cancer is being studied extensively because of its high mortality and morbidity rates and its association with habits such as alcoholism and smoking as the major risk factors in its etiology. Alcohol consumption and smoking, which are considered risk factors for *S. anginosus* infection, may contribute to the development of both head and neck cancer and esophageal cancer, throat and head cancer [16]. Free radical damage to DNA is one of the primary etiologic processes of cancer development. Nitric oxide (NO) production by macrophages can be triggered by a number of antigens and microorganism components, including lipopolysaccharide (LPS), peptidoglycans, lipoteichoic acids, and carbohydrate antigens [17]. Approximately only 15% of oral carcinoma cases are not found to be associated with any adverse habits. Recent studies have shown that bacteria may act as a mediator in the etiological association with oral cancer. Additionally, variations in bacterial microflora have been found in chain smokers and alcohol addicts. *Streptococcus anginosus* has been detected in tumor samples from patients with oropharyngeal, oesophageal, and gastric cancer using species-specific molecular methods [18]. Nagy et al. found that the surface of developed tumors showed an increased number of some of the oral microbial species in comparison to healthy tissue sites. Although the link between the pathogenic bacterium and oral carcinomas cannot be negated, there is little understanding of the exact role of these species in the development of oral cancer. Bacterial groups referred to as "commensals" are the groups of bacteria living in harmony with the host defense system. The most predominant genus in the healthy oral microbiome is streptococcus. Among the less frequently found species are Prevotella, Veillonella, Actinomyces, and Neisseria. Dominating an individual’s oral cavity are the groups Fusobacterium, Treponema, Lactobacterium, Capnocytophaga, Porphyromonas, Eubacterium, Eikenella, Peptostreptococcus, Haemophilus, and Propionibacterium. Commensal bacterial species are known to compete for nutritional supply and receptor sites with all the exogenous microbes, thus making them an essential part of host immunity. Additionally, these bacterial species produce antagonists, namely bacteriocins that can cause an inhibition of certain susceptible bacterial species that are extrinsic in origin. A persistent expression of major histocompatibility complex (MHC) class II molecules on cells such as macrophages is brought about by lipopolysaccharides being released by the microbiota. This leads to cross‑protective antibody production. Hence, the resident microflora is able to serve as a barrier to exogenous bacterial species and contribute to the host defense mechanisms.

Role of alcohol in carcinogenesis, in the presence of normal oral microflora

**Figure 1:**
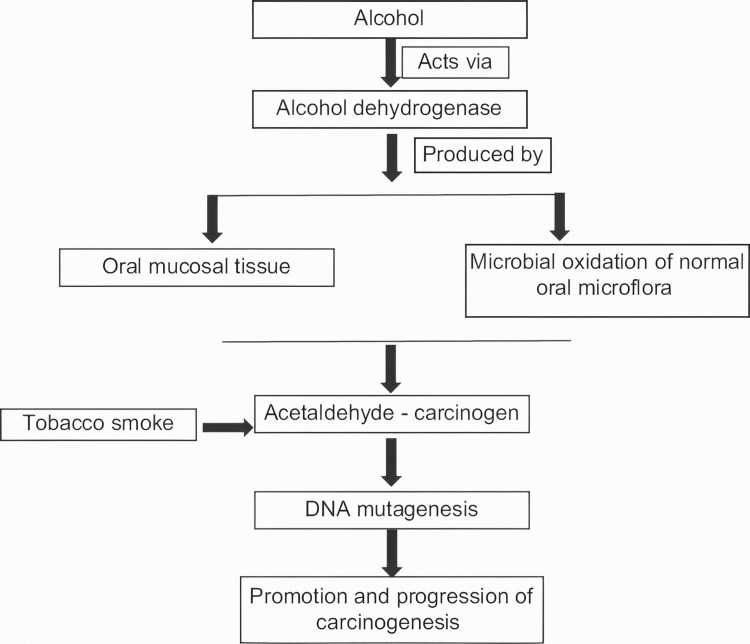
Interrelationship of oral microflora, alcohol, and oral carcinogenesis The author has taken the figure from the source [1]

As seen in Figure 1, alcohol mainly contributes to the process of carcinogenesis by the production of the enzyme alcohol dehydrogenase, the effect of which is further demonstrated. Alcohol dehydrogenase, an enzyme that can convert ethanol to acetaldehyde is abundantly produced by the bacterium Neisseria, which is a regular inhabitant of the oral microbiome. ACH is a well-established carcinogen. A definite trend in aerobic conditions was evident in the saliva of "high" and "low" acetaldehyde producers after the microbiological examination. Aerobe total counts dramatically rose among "high" producers. Aerobic species such as *Streptococcus salivarius*, hemolytic *Streptococcus viridans* var., Corynebacterium spp., Stomatococcus spp., and yeasts were strongly linked to greater ACH production. Among the patients who produced more ACH, yeasts were not only more frequently identified but were also found in higher concentrations. "High" producers had somewhat higher overall anaerobic numbers, but there was no relationship between any particular bacterial species and ACH production [19].

Srinivas Prasad et al. demonstrated how the normal oral microflora produces ACH in ethanol incubation {5]. Hence, the resident microflora can lead to carcinogenesis by the conversion of ethanol into a genotoxic metabolic product, ACH. Several research works have been conducted to support this concept, and microorganisms such as Gram-positive aerobic bacteria, yeasts, and streptococci have all been linked to ACH synthesis to date. ACH can be derived in substantially higher levels from ethanol oxidation by the local oral microbiota, in addition to being produced by mucosal alcohol dehydrogenases. ACH is categorized as a group I carcinogen by the International Agency for Research on Cancer. By generating DNA damage, ACH can cause a variety of mutagenic consequences, including but not limited to DNA adducts, DNA crosslinking, aneuploidy, and chromosomal abnormalities. According to Homann et al., smokers can have up to seven times greater salivary ACH concentrations than non-smokers, even after moderate alcohol consumption. Tobacco smoke also contains ACH, which may lead to the selection of microorganisms with a high rate of ACH metabolism and higher tolerance after repeated exposure [19]. Chronic smoking causes changes in the oral microflora, resulting in increased ACH synthesis. This suggests that oral cancer patients' saliva has a higher chance of microbial flora survival and replication. Thus, in the etiology of oral cancer, the oral bacterial flora can work in tandem with major risk factors including alcohol abuse and smoking. Because the oral microbiota is one of the most important sources of local ACH, increasing oral hygiene in these people can reduce the amount of ACH produced.

Role of oral hygiene practices in carcinogenesis, in the presence of normal microflora

**Figure 2:**
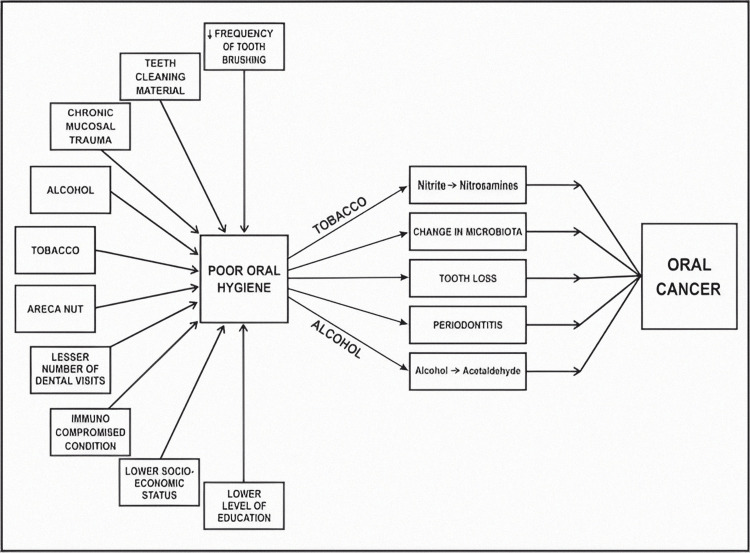
Poor oral hygiene and its effects leading to oral cancer The author has taken the figure from the source [22]

Variations in the oral environment can cause a disruption in the active balance between the various microbial groups. The overexpression of virulence factors and pro-inflammatory characteristics by an initial "passenger" microbiome within the tumor microenvironment is believed to contribute to the tumor's growth [20]. With the onset of any disease in the oral cavity, there occurs a shift in the oral microbial balance that favors non-resident pathogenic microbial species. Periodontal disorders are characterized by a change in the bacterial flora of the gums, as well as an inflammatory response that can be pathogenic. An important periodontal bacterium called *Porphyromonas gingivalis* is epidemiologically linked to both OSCC and pancreatic cancer. *P. gingivalis* can block chemically induced apoptosis and speed up cell cycle progression in primary cultures of gingival epithelial cells, traits that are associated with the encouragement of carcinogenesis. Patients with oral cancer have been shown to have *P. gingivalis* in their oral cavity and on their OSCC surfaces in clinical samples [21]. In Figure 2, various factors that can lead to poor oral health and how this further disposes the tissues for the development of cancer are shown.

Poor oral health has also been proposed as a factor in facilitating the conversion of ethanol to carcinogenic ACH via bacterial enzyme metabolism. Mager et al. studied bacterial counts of salivary samples from oral cancer patients and compared them with the microflora residing in the oral cavities of healthy individuals. The most notable changes were seen in counts of species such as *Prevotella melaninogenica*, *S. mitis,* and *Capnocytophaga gingivalis* among the patients having cancer. According to Sasaki et al., dental plaque contains *Streptococcus anginosus*, a bacterium that can cause an infection of the oral mucosa, which can lead to the initiation of carcinogenesis in the infected tissues. Chronic inflammation induction is regarded as a different possible pathway in which *Porphyromonas gingivalis* takes a role in the development of oral cancer. It is well recognized that chronic inflammation considerably contributes to the formation of the OSCC, mostly through modifying its microenvironment with the use of cytokines and chemokines [23]. When compared with the healthy mucosa, OSCC lesions show elevated levels of Fusobacterium and Porphyromonas. Hence it can be assumed that effective oral hygiene has a significant role in maintaining the integrity of the tissues that are prone to undergo a cancerous transformation and specific care should be taken to avoid carelessness in such patients regarding regular and effective oral healthcare.

Role of diet and nutrition in oral carcinogenesis, in the presence of normal microflora

Tobacco smoking and alcohol consumption are important factors associated with the development of carcinomas. However, the presence of these two factors solely is unable to explain the occurrence of carcinomas. Alcohol is a cocarcinogen that can act as a cancer-causing agent alone or in conjunction with tobacco as a synergistic agent. Viral infections caused by poor oral hygiene, contact with cancer-causing substances, exposure to UV and ionizing radiation, radioactivity, socioeconomic status, and persistent trauma are regarded as additional risk factors [24].

Dietary habits and the levels of micronutrients present within the body play an important role in maintaining the tissues in their normal state in terms of their integrity, functioning, and defenses. A normal healthy diet should consist of sufficient proportions of cereals, pulses, fruits, vegetables, nuts, dairy products, etc. so as to incorporate the appropriate amounts of fats, protein, carbohydrates, vitamins, and minerals. A variety of micronutrients play an irreplaceable role in the maintenance of the overall integrity of the tissues. The body’s immune system also depends heavily on the levels of dietary nutrients present within the body. In their absence, the bacteria have greater chances of invading the cells and altering their immunochemistry, thereby resulting in increased chances of the development of carcinoma. Hence poor dietary status coupled with a resident microflora can lead to an increased propensity for carcinogenesis, and the presence of risk factors such as alcoholism and smoking can adversely hamper the cells’ ability to resist malignant transformation.

Oral rinse samples taken before and after treatment were compared, and the results revealed that numerous bacteria differ between the two periods. Additionally, compared to the baseline pretreatment sample, some bacterial species showed altered abundance trends that were considerably different. Six months after surgery, the abundance of Streptococcus, Rothia, and Gemella increased whereas Fusobacterium, Capnocytophage, Prevotella, Alloprevotella, and Leptotrichia declined. These patterns point to a post-treatment shift in favor of bacterial populations that resemble healthy controls. It also reflects the previously observed reverse pattern of an increase in the relative abundance of Fusobacterium from healthy controls to patients with premalignant oral cavity lesions and lastly to patients with OSCC [25].

## Conclusions

Oral carcinoma is a multi-factorial disease. A variety of microbiological agents can contribute to the progression of carcinogenesis in the presence of definitive risk factors such as alcoholism and smoking. Despite extensive research in this area, a definitive link between cancer and microbiota has not yet been established. Various hypotheses have been formulated to understand and explain the contributory role of these agents.

## References

[1] Gaonkar PP, Patankar SR, Tripathi N (2018). Oral bacterial flora and oral cancer: The possible link?. J Oral Maxillofac Pathol.

[2] Yang CY, Yeh YM, Yu HY (2018). Oral microbiota community dynamics associated with oral squamous cell carcinoma staging. Frontiers in microbiology.

[3] Vogtmann E, Goedert JJ (2016). Epidemiologic studies of the human microbiome and cancer. Br J Cancer.

[4] Sixou JL, de Medeiros-Batista O, Bonnaure-Mallet M (199632). Modifications of the microflora of the oral cavity arising during immunosuppressive chemotherapy. Eur J Cancer B Oral Oncol.

[5] Srinivasprasad V, Dineshshankar J, Sathiyajeeva J ( 2015). Liaison between micro-organisms and oral cancer.. J. Pharm. Bioallied Sci..

[6] Sakamoto H, Naito H, Ohta Y (1999). Isolation of bacteria from cervical lymph nodes in patients with oral cancer. Arch Oral Biol.

[7] Marttila E, Uittamo J, Rusanen P, Lindqvist C, Salaspuro M, Rautemaa R (2013). Acetaldehyde production and microbial colonization in oral squamous cell carcinoma and oral lichenoid disease. Oral Surg Oral Med Oral Pathol Oral Radiol.

[8] Hooper SJ, Crean SJ, Fardy MJ, Lewis MA, Spratt DA, Wade WG, Wilson MJ (2007). A molecular analysis of the bacteria present within oral squamous cell carcinoma. J Med Microbiol.

[9] Butel JS (2000). Viral carcinogenesis: revelation of molecular mechanisms and etiology of human disease. Carcinogenesis.

[10] Sanjaya PR, Gokul S, Gururaj Patil B, Raju R (2011). Candida in oral pre-cancer and oral cancer. Med Hypotheses.

[11] Tateda M, Shiga K, Saijo S (2000). Streptococcus anginosus in head and neck squamous cell carcinoma: implication in carcinogenesis. Int J Mol Med.

[12] Mager DL, Ximenez-Fyvie LA, Haffajee AD, Socransky SS (2003). Distribution of selected bacterial species on intraoral surfaces. J Clin Periodontol.

[13] Sakamoto H, Sasaki J, Nord CE (1999). Association between bacterial colonization on the tumor, bacterial translocation to the cervical lymph nodes and subsequent postoperative infection in patients with oral cancer. Clin Microbiol Infect.

[14] Lax AJ, Thomas W (2002). How bacteria could cause cancer: one step at a time. Trends Microbiol.

[15] Martin MV, Al-Tikriti U, Bramley PA (1981). Yeast flora of the mouth and skin during and after irradiation for oral and laryngeal cancer. J Med Microbiol.

[16] Rajeev R, Choudhary K, Panda S, Gandhi N (2012). Role of bacteria in oral carcinogenesis. South Asian J Cancer.

[17] Sasaki M, Yamaura C, Ohara-Nemoto Y (2005). Streptococcus anginosus infection in oral cancer and its infection route. Oral Dis.

[18] Hooper SJ, Crean SJ, Lewis MA, Spratt DA, Wade WG, Wilson MJ (2006). Viable bacteria present within oral squamous cell carcinoma tissue. J Clin Microbiol.

[19] Homann N, Tillonen J, Meurman JH (2000). Increased salivary acetaldehyde levels in heavy drinkers and smokers: a microbiological approach to oral cavity cancer. Carcinogenesis.

[20] Nezar N. Al-Hebshi1 & Wenche S (2019). The microbiome of oral squamous cell carcinomas: a functional perspective. Current Oral Health Reports.

[21] Inaba H, Amano A, Lamont RJ, Murakami Y (2015). Involvement of protease-activated receptor 4 in over-expression of matrix metalloproteinase 9 induced by Porphyromonas gingivalis. Med Microbiol Immunol.

[22] Mathur R, Singhavi HR, Malik A, Nair S, Chaturvedi P. (2019). Role of Poor Oral Hygiene in Causation of Oral Cancer-a Review of Literature.. Indian J Surg Oncol..

[23] Vyhnalova Vyhnalova, T.; Danek, Z.; Gachova, D D (2021). The Role of the Oral Microbiota in the Etiopathogenesis of Oral Squamous Cell Carcinoma. Microorganisms10.3390/microorganisms9081549.

[24] Bolz J, Dosá E, Schubert J, Eckert AW (2014). Bacterial colonization of microbial biofilms in oral squamous cell carcinoma. Clin Oral Investig.

[25] Chan JYK, Ng CWK, Lan L (2021). Restoration of the Oral Microbiota After Surgery for Head and Neck Squamous Cell Carcinoma Is Associated With Patient Outcomes. Front. Oncol.

